# Supplementation of Japanese elite athletes for the Paris 2024 Olympic games: differences according to nutritional support experience

**DOI:** 10.3389/fspor.2026.1892171

**Published:** 2026-07-30

**Authors:** Akiho Shinagawa, Kanae Myoenzono, Eri Takai, Hiroka Ohno, Keiko Namma-Motonaga, Etsuko Kamihigashi, Kazuyuki Kamahara, Akiko Kamei

**Affiliations:** 1 Department of Sports Medicine, Japan Institute of Sports Sciences, Japan High Performance Sport Center, Tokyo, Japan; 2 Faculty of Sports Sciences, Waseda University, Saitama, Japan

**Keywords:** anti-doping, hydration, nutritional support, Olympic athletes, supplementary foods, supplements

## Abstract

**Background:**

Supplements are popular among athletes. To prevent anti-doping rule violations, athletes, registered dietitians, and support professionals must assess the safety, efficacy, and necessity of supplements. However, the relationship between experience with receiving nutritional support and supplement intake has not yet been examined.

**Objective:**

In this study, we aimed to investigated differences in supplement use according to nutritional support experience using the Australian Institute of Sport classification system.

**Methods:**

A self-administered questionnaire was completed by 800 athletes representing Japan at the Paris 2024 Olympic Games (including candidates). The survey assessed supplement intake over the past year, nutritional support experience, and knowledge and practices regarding supplements and hydration.

**Results:**

Overall, 92.3% of athletes used supplements, with a higher percentage among those who had nutritional support experience (*p* < 0.01). Among athletes who consumed group A sports foods (evidence-based supplements), the intake rates were higher among those with nutritional support experience than among those without nutritional support experience (*p* < 0.01). Even after adjusting for age and sex, nutritional support experience was associated with total supplements and sports foods intake.

**Conclusion:**

Nutritional support experience appears to be associated with the intake of supplements, particularly sports foods. Although limited to associations, these findings highlight the need for dietitians to collaborate with multidisciplinary staff when providing athletes with comprehensive, evidence-based education on supplement efficacy and safety.

## 1 Introduction

Dietary supplements are commonly consumed by athletes across a wide range of ages and competitive levels. According to the International Olympic Committee (IOC), a supplement is defined as “a food, food component, nutrient, or non-food compound that is purposefully ingested in addition to the habitually consumed diet with the aim of achieving a specific health and/or performance benefit” (1). In our previous studies, we found that Japanese athletes (including candidates) representing Japan at the summer Olympics take supplements to promote recovery from fatigue and improve performance (2–4). Evidence also suggests that supplement use may be effective in certain situations, such as when specific nutrients are deficient due to dietary characteristics (e.g., allergies or religious practices) or when consuming adequate food before, during, or after exercise proves difficult (5). However, supplement use carries a risk of violating anti-doping rules (6). The World Anti-Doping Agency (WADA) applies the principle of strict liability. Under the WADA code, “strict liability” means that each athlete bears personal responsibility for ensuring that prohibited substances do not enter their body (7–9). Therefore, athletes must adopt measures to reduce the risk of anti-doping rule violations associated with supplement use. Anti-doping rule violations also apply to athlete support staff. Accordingly, dietitians and other staff who support athletes must thoroughly evaluate the safety and effectiveness of supplements.

The Australian Institute of Sport (AIS) (10) categorizes supplements into four groups (A, B, C, and D) based on scientific evidence and product safety. Group A comprises supplements with strong scientific support for use in sport-specific situations, such as sports foods, medical supplements, and performance supplements (e.g., caffeine, creatine). Group B consists of supplements for which scientific evidence is emerging but remains insufficient, warranting further investigation. Group C includes supplements that lack scientific evidence supporting a benefit for athletes or have not been studied sufficiently to allow an informed conclusion. Group D comprises supplements associated with a high risk of contamination with prohibited substances or with a likelihood of producing a positive doping test result (10). Hereafter, this classification is referred to as the ABCD classification system.

Studies (11–22) have reported the supplement intake status of athletes using the ABCD classification system, mainly investigating differences according to sex, competitive level, and playing position. A higher rate of supplement intake was observed among male athletes (17, 18) and athletes competing at higher levels (12, 16, 18), and position-specific differences in supplement use was reported among soccer players (11). In addition, the percentage of athletes who identified a dietitian as their main source of information on supplement use was found to be significantly higher at higher competitive levels than at lower levels (18). In contrast, our previous study demonstrated that many athletes use supplements before undergoing evaluation by a physician or dietitian to assess need, and that some athletes consume supplements without confirming the presence or absence of substances prohibited by the WADA (4). The IOC consensus statement (1) recommends that decisions regarding supplement use be made based on medical diagnosis and nutritional evaluation by a dietitian and that dietitians supporting athletes provide guidance on supplement use. Such guidance includes information on supplement safety and effectiveness, which form the basis for the ABCD classification system. Effective athlete support also requires collaboration with other experts, including physicians, sports pharmacists, coaches, and trainers, to prevent anti-doping rule violations. Dietitians play an indispensable role in clinical practice; their proactive guidance is vital not only for optimizing athletic performance but also for curbing the unnecessary intake of supplements that lack scientific evidence.

Considering the role of dietitians in athlete support, nutritional support provided by dietitians, defined in this study as including nutrition seminars and ongoing nutritional counseling, seems closely related to athletes' supplement use. A study reported that athletes who receive nutritional counseling are more likely to use several supplements, such as vitamin D and isotonic sports drinks (23). However, that study investigated supplement use only over the preceding 4 weeks, raising concern about potential misestimation of habitual intake. Assessment of supplement use over a longer period may provide a more accurate representation of intake patterns (23). In addition, although several studies (2–4) have reported on the prevalence of supplement use among Japanese elite athletes, to our knowledge, no previous study has applied the ABCD classification system to a large cohort of Japanese elite athletes. This structured approach enables a clearer distinction between evidence-based supplements (group A) and supplements with limited or no evidence (group C), providing a more nuanced understanding of the quality of supplement choices, compared with previous descriptive reports. Furthermore, this framework allows for a systematic evaluation of supplement use from an evidence-based and anti-doping perspective. Professional nutritional support is expected to enhance athletes' knowledge about supplement, thereby facilitating informed choices. Conversely, athletes frequently rely on information from immediate people, such as coaches, peers, and family members (24). Consequently, athletes without nutritional support may rely on their own judgment based on these subjective sources when choosing supplements. Therefore, the aim of this study was to characterize the supplement use of Japanese elite athletes over the previous year and to determine whether supplement intake differs according to experience with receiving nutritional support. The hypotheses were as follows:

 Hypothesis 1. Athletes with nutritional support experience would be more likely to take supplements, compared with those without experience.

 Hypothesis 2. Athletes with nutritional support experience would show higher intake of group A supplements, whereas those without experience would show higher intake of group C supplements.

## 2 Materials and methods

### 2.1 Participants

This cross-sectional, questionnaire-based study examined supplement use among Japanese elite athletes. Participants consisted of 800 Japanese national team athletes or candidates for the 33rd Olympic Games (Paris/2024) who underwent routine medical checkups at the Japan Institute of Sports Sciences between November 1, 2023, and July 26, 2024.

### 2.2 Ethics

The Ethics Committee of the Japan Institute of Sports Sciences reviewed and approved the study (approval number: 2024-013), which was conducted in accordance with the tenets of the Declaration of Helsinki. All participants provided written informed consent before completing the questionnaires during the medical checkups. The website (https://www.jpnsport.go.jp/hpsc/Portals/0/resources/jiss/pdf/optout/2024-013.pdf) provided detailed information explaining the study to the athletes. This website included optout information for individuals who did not agree to the use of their data and allowed participants to withdraw consent at any time. For participants younger than 18 years, a legal representative was given the opportunity to decline participation in the study.

### 2.3 Procedure and questionnaire

As part of the medical checkup, participants completed a self-administered questionnaire using an application (NOBORI; PSP Corporation, Tokyo, Japan) that assessed nutritional knowledge, experiences, behaviors, and supplement use during the previous year. This questionnaire was developed based on a baseline tool employed in similar surveys targeting Japanese elite athletes (4). For this current study, the items of the questionnaire were updated and refined by registered dietitians to better capture the required nutritional behaviors. Although the questionnaire was originally developed for clinical use in medical checkups to assess athletes' dietary supplement use and related nutritional behaviors, and was not formally validated for research purpose, several measures were taken to ensure precision and consistency of the collected data. Specifically, trained registered dietitians reviewed the questionnaire responses, and each participant underwent an individual inter-view to clarify omissions or errors, thereby ensuring the accuracy and reproducibility of the responses. In addition to collecting data regarding age, sex, and sport, the questionnaire included the following items:

1. Have you ever received nutritional support, such as seminars or consultations with a nutritionist?
2. Are you familiar with the significance (role and use) of supplementary foods?
3. Do you consume supplementary foods when you need?
4. Do you know when and how much hydration is required during training sessions?
5. Do you believe you achieve adequate hydration?
6. Do you regularly consume sports drinks, sports jellies/gels, or sports bars?
7. Do you take any supplements?
8. If you answered “Yes” to question 7, please provide the following information: (8-1) product name; (8-2) manufacturer.

The supplements reported by the participants were classified by a registered dietitian based on the ABCD classification system. Participants who consumed sports drinks, sports jellies/gels, or sports bars, as included in question 6 of the questionnaire, were classified as consumers of group A sports foods, based on the AIS supplement classification.

### 2.4 Statistical analysis

The Kolmogorov–Smirnov test was used to assess normality of the variables. The Mann–Whitney U test was performed to compare age differences between participants with and those without nutritional support experience. Categorical variables were compared using the chi-square (*χ*^2^) test, as appropriate. Based on a previous study (25) reporting differences in supplement use according to age and sex, logistic regression analysis was performed to evaluate the association between supplement use and nutritional support experience, adjusting for age and sex. Odds ratios (ORs) and 95% confidence intervals (CIs) were calculated. The dependent variable was supplements use (0 = no supplement intake, 1 = supplement intake), and the independent variables were nutritional support experience (0 = no nutritional support experience, 1 = nutritional support experience), sex (0 = female, 1 = male), and age. Descriptive statistics were presented as medians (interquartile ranges) for quantitative variables and as percentages for qualitative variables. All statistical tests were two-tailed, and the significance level was set at *p* < 0.05. Statistical analyses were performed using Statistical Package for Social Sciences (SPSS) version 29 (IBM Corporation, NY, USA).

## 3 Results

### 3.1 Participant characteristics

No participants withdrew from the study, leaving data from 800 athletes (414 male, 386 female) for analysis. The participants represented 31 of the 32 sports held at the 33rd Olympic Games (Paris/2024), with the exclusion of Taekwondo. Participant ages ranged from 14 to 48 years. Nutritional support experience was reported by 86.9% of the athletes (*n* = 695). Athletes with nutritional support experience had a median age of 26.0 (23.0–29.0) years, whereas those without nutritional support experience had a median age of 25.0 (21.0–28.0) years, representing a significant difference (*p* = 0.020). The male-to-female ratio did not differ significantly between athletes with and those without nutritional support experience (*p* = 0.329).

### 3.2 Total supplements intake ratio by nutritional support experience

Table 1 shows the overall percentage of supplement intake among participants. During the past year, 92.3% of the participants reported consuming one or more supplements. Across all participants, the mean number of supplements consumed per athletes was 2.3 (with different flavors of the same product counted as a single supplement). The distribution of supplement intake according to the ABCD classification system indicated the highest intake proportion for group A supplements (91.4%) followed by group C supplements (43.1%), whereas group B supplements showed the lowest intake proportion (9.1%). Group A sports foods showed the highest intake rate. No athletes reported consuming supplements categorized as tastants in group C. Group C includes “the rest” referring to substances that cannot be classified into group A, B, or D (10). In this study, these included French maritime pine bark extract, glutamine, arginine, and glycine. Stratified analysis by nutritional support experience revealed a significantly higher prevalence of supplement use among athletes with nutritional support experience than among athletes without such experience (93.7% vs. 82.9%, *p* < 0.001). In addition, the intake proportion of group A supplements differed according to nutritional support experience (*p* = 0.003). Specifically, athletes with nutritional support experience reported a higher consumption rate of group A sports foods, compared with athletes without such experience (*p* < 0.001).

**Table 1:** Supplement intake ratio across groups defined by the Australian institute of sport according to nutritional support experience.

Supplement category		Total (%) (*n* = 800)	Nutritional support experience (%)			
			Yes (*n* = 695)	No (*n* = 105)	*p*	*φ*
Total supplements		92.3	93.7	82.9	<0.001	0.137
Group A supplements	Total	91.4	92.5	83.8	0.003	0.105
	Sports foods	89.9	91.4	80.0	<0.001	0.127
	Medical supplements	23.3	23.9	19.0	0.274	0.039
	Performance supplements	12.4	12.4	12.4	0.998	0.000
Group B supplements	Total	9.1	9.1	9.5	0.879	−0.005
	Food polyphenols	0.3	0.3	0.0	0.755	0.019
	Antioxidants	4.4	4.3	4.8	0.496	−0.007
	Tastans	0.0	0.0	0.0	—	—
	Other	5.3	5.3	4.8	0.810	0.009
Group C supplements	Total	43.1	44.5	34.3	0.050	0.069
	Named products	36.4	37.4	29.5	0.117	0.055
	The rest	17.3	17.8	13.3	0.254	0.040

Values are presented as percentage. None of the athletes reported intake of group D supplements; therefore, this group was excluded from the table.

*p* values obtained using *χ*^2^ test for categorical variables.

Table 2 shows the results of a logistic regression analysis with supplement use as the dependent variable and experience with nutritional support, age, and sex as independent variables. After adjusting for age and sex, experience with nutritional support was found to be significantly associated with total supplement intake. Athletes with nutritional support experience demonstrated a higher OR for supplements use than did athletes without such experience (OR = 3.11, 95% CI = 1.72–5.65, *p* < 0.001). Furthermore, even after adjusting for age and sex, a significant association was found between the intake of group A supplements and nutritional support experience (OR = 2.42, 95% CI = 1.33–4.38, *p* = 0.004). Of the group A supplements, sports foods showed a significant association with nutritional support experience (OR = 2.68, 95% CI = 1.54–4.64, *p* < 0.001).

**Table 2:** Supplement intake ratio in each group as defined by the Australian institute of sport, based on experience with nutritional support, adjusted for age and sex.

Supplement category		OR (95% CI)	*p*
Total supplements		3.11 (1.72–5.65)	<0.001
Group A supplements	Total	2.42 (1.33–4.38)	0.004
	Sports foods	2.68 (1.54–4.64)	<0.001
	Medical supplements	1.31 (0.78–2.19)	0.315
	Performance supplements	1.01 (0.53–1.89)	0.988
Group B supplements	Total	0.85 (0.42–1.73)	0.658
	Antioxidants	0.85 (0.32–2.27)	0.751
	Other	0.98 (0.37–2.59)	0.988
Group C supplements	Total	1.48 (0.96–2.28)	0.077
	Named products	1.37 (0.88–2.15)	0.166
	The rest	1.36 (0.74–2.47)	0.320

Binary logistic regression analysis. When supplement intake was examined according to experience with nutritional support (Table 1), food polyphenols and tastants, which showed 0% prevalence, were excluded from this table.

OR, odds ratio; 95% CI, 95% confidence interval.

### 3.3 Knowledge and practices related to supplementary foods and hydration among athletes consuming sports foods

**Figure 1:**
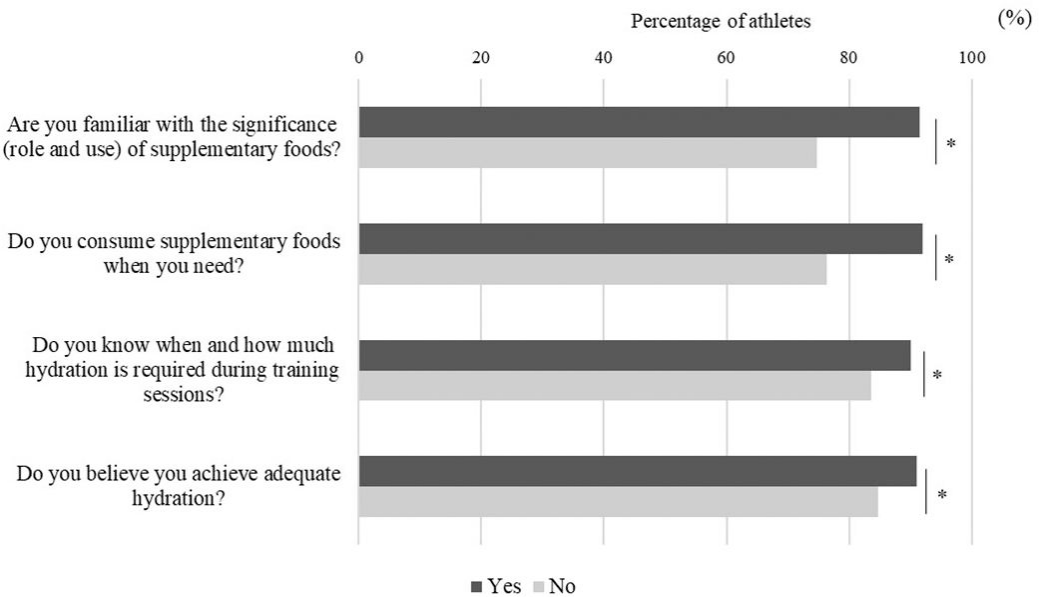
Knowledge and actual status of supplementary foods and hydration among athletes who consume sports foods. Responses among sports foods users (*n* = 719). *p < 0.01 as per *χ*^2^ test for categorical variables.

Figure 1 shows the knowledge and practices related to supplementary foods and hydration among athletes who consumed sports foods. A significantly higher proportion of them reported adequate knowledge of supplementary foods and hydration and the ability to consume supplementary foods and hydration as needed (*p* < 0.001). Athletes who did not consume sports foods showed no significant differences in the knowledge or practices regarding supplementary foods and hydration.

### 3.4 Knowledge and practices regarding supplementary foods and hydration

Table 3 presents the percentage of athletes who possessed knowledge of supplementary foods and hydration and the percentage who were able to consume supplementary foods and hydration as needed, stratified by nutritional support experience. Among athletes with knowledge of supplementary foods and hydration, a significantly higher percentage reported having nutritional support experience compared with having no such experience (*p* < 0.001). A similar trend was observed among athletes who were able to consume supplementary foods and hydration as needed (*p* < 0.001).

**Table 3:** Percentage of athletes who reported having knowledge of supplementary foods and hydration and those who reported being able to consume supplementary foods and hydration as needed.

Item	Total (%) (*n* = 800)	Nutritional support experience (%)		
		Yes (*n* = 695)	No (*n* = 105)	*p*
Have knowledge of supplementary foods	90.1	94.7	60.0	<0.001
Able to take the supplementary foods as needed	86.3	91.4	52.4	<0.001
Have knowledge of hydration	85.5	89.2	61.0	<0.001
Hydration can be provided as needed	82.0	85.5	59.0	<0.001

Values are presented as percentage.

*p* values obtained using *χ*^2^ test for categorical variables.

## 4 Discussion

From the total surveyed sample of Japanese elite athletes, 92.3% reported consuming supplements. Athletes with nutritional support experience demonstrated a significantly higher rate of supplement intake, particularly sports foods, compared with athletes without such experience. However, for group C supplements, which have a lower level of evidence, borderline significance was observed when comparing the proportion of athletes taking supplements with and without experience of nutritional support. After adjusting for age and sex, a significant association was observed between nutritional support experience and the intake of sport foods. Specifically, athletes with such experience had higher odds of consuming sports foods. For group C supplements, a clear association between their intake and nutritional support experience could not be established, even after adjusting for age and sex. Although the results of this study are limited to identifying associations and do not establish causality, these findings suggest that support staff may need to communicate more clearly and assertively with athletes regarding the efficacy and safety of supplements. Dietitians who support athletes should collaborate with doctors, pharmacists, and other support staff to provide comprehensive education on supplement use.

One aim of this study was to determine the proportion of supplement users among Japanese elite athletes; the survey revealed that 92.3% of the athletes had taken one or more supplements in the past year. A study conducted over the past 5 years, using the ABCD classification system to investigate supplement intake among athletes, reported supplement intake rates ranging from 46.9% to 93.8% (11–22). The results of this study were relatively high, within this range. Several studies have shown that athletes competing at higher levels are more likely to take supplements (12, 13, 16, 18, 22). For example, 96.2% of triathlon athletes competing in international competitions, a competitive level comparable with that of the participants in this study, reportedly take supplements (14). The participants in this study consisted of Japanese Olympic athletes or candidates, a group competing at a high level, which likely contributed to the high rate of supplement intake observed.

Of athletes with and those without nutritional support experience, a higher percentage of the former group reported taking supplements. This result supported the study hypothesis 1. Athletes with nutritional support experience were also older than athletes without such experience (26.0 years vs. 25.0 years, *p* = 0.020). Sebastiá-Rico et al. (11) reported that older athletes had a higher rate of supplement intake. In this study, age may have contributed to the higher rate of supplement intake observed among athletes with nutritional support experience. Conversely, as shown in Table 2, a significant association was found between supplement intake and nutritional support experience, even after adjusting for age and sex (*p* < 0.001). Although nutritional support experience may have influenced supplement use, reverse causation cannot be ruled out. Given that the participants in this study were high-level athletes, it is possible that those with greater awareness of performance and conditioning were already using supplements and were therefore more likely to seek nutritional support. Previous studies have suggested that supplement use is more prevalent among athletes with higher performance orientation and competitive level (1, 25, 26). In addition, supplement use among athletes has been reported to vary depending on the type of sport, training load, competition level, age, and sex (26). As our study population consisted of national-level elite athletes, disclosing the exact number of participants per discipline alongside other demographic data could potentially lead to the identification of specific individuals. To maintain strict confidentiality and protect the anonymity of these elite competitors, we prioritized data aggregation. This approach ensures ethical protection and statistical stability; however, it may obscure discipline-specific nuances. Although we adjusted for age and sex, other factors such as type of sport, training volume, and access to resources were not considered and may have influenced the observed association. Also, notably, the frequency and type of nutritional support varied widely within this study, ranging from continuous individual counseling to one-time participation in a nutrition seminar. Although these different levels of intervention likely exert distinct influences on athletes' behavior and supplement literacy, we analyzed all intervention levels under a single exposure factor, preventing the examination of the specific effects of each level. Consequently, the potential impact of continuous, individualized nutritional support might have been underestimated. Conversely, a one-time seminar attendance may not have provided sufficient knowledge retention to alter long-term supplementation patterns. Future studies should classify nutritional support based on intervention characteristics, such as duration, frequency, and specific content, to clarify how varying levels of support influence supplement use patterns.

Athletes with nutritional support experience consumed group A sports foods at a significantly higher rate, compared with those without such experience (91.4% vs. 80.0%, *p* < 0.001). Even after adjusting for age and sex, this association remained significant (*p* < 0.001). Group A sports foods comprised items used as supplementary foods, such as sports gels and bars, as well as foods consumed for hydration, such as sports drinks and electrolyte supplements. Figure 1 shows that athletes who consumed sports foods possessed knowledge about supplementary foods and hydration and reported appropriate consumption practices. Furthermore, Table 3 shows that more athletes with nutritional support experience had knowledge about supplementary foods and hydration and were able to consume foods appropriately. During nutritional support, many opportunities arise to communicate the importance of supplementary foods for recovery and conditioning after training or competition. When studies indicate the effectiveness of sports foods, such as sports gels and sports drinks (27–29), these options can be recommended for supplementary food intake and hydration. These findings may partially explain the relationship between nutritional support experience and the rate of sports food consumption. However, some athletes may have independently acquired knowledge about supplementary foods and hydration, conducted their own research, and consumed supplements accordingly. Consequently, the present survey design does not allow causal relationships to be determined.

We examined the differences in the percentage of athletes taking group C supplements according to nutritional support experience, and the *p*-value was 0.050. After adjusting for age and sex, intake of group C supplements and nutritional support experience showed a positive association (OR = 1.48); however, the 95% CI included 1.0 (95% CI: 0.96–2.28, *p* = 0.077), indicating borderline significance. Therefore, it is difficult to draw definitive conclusions regarding this association based solely on the results of the present study. A study showed that amino acid supplements containing branched-chain amino acids (BCAAs) ranked among the most commonly used supplements by Japanese elite athletes (4). BCAAs are also the most commonly used supplements by elite athletes in Spain (30). In addition, in Japan, supplements primarily composed of BCAAs were provided by the official sponsors for the 33rd Olympic Games (Paris/2024). In this study, among athletes who reported consuming supplements classified as “named products” in group C, including BCAAs, 52.9% listed names of products that had been provided to them free of charge. The lack of a clear difference in group C supplement intake rates based on nutritional support experience may be partly explained by the general popularity of BCAAs and potential influence of sponsorships.

Although BCAAs are popular among athletes, no consensus exists regarding the effectiveness of BCAA supplementation on sports performance (31). A systematic review (32) indicated that research on dietary supplements in athletes has predominantly focused on protein, creatine, caffeine, and *β*-alanine, whereas evidence for other commonly used supplements, such as BCAAs, remains relatively limited or inconsistent. The IOC overview also does not mention the topic of BCAAs (1). In the ABCD classification system, BCAA supplements are categorized in group C, which has a low level of evidence (10). The IOC recommends that supplement use be based on confirmation through a physician's diagnosis or a nutritional evaluation conducted by a registered dietitian (1). However, a study (4) showed that more than half of elite athletes use supplements without evaluation by a physician or registered dietitian. Athletes in that study may have similarly selected supplements independently, regardless of whether they received nutritional support. Physicians and registered dietitians should ensure that athletes possess sufficient knowledge about supplement use and should emphasize the efficacy and safety of any supplements under consideration. These findings provide practical insights for designing optimized nutrition education programs for athletes. The high prevalence of supplement use observed in this study (92.3%) underscores the ubiquity of supplements among elite athletes; however, a high usage rate does not necessarily equate to appropriate or evidence-based practice. In fact, a considerable proportion of our participants used group C supplements, for which scientific evidence on efficacy remains limited. This discrepancy highlights a critical gap between supplement consumption and supplement literacy. Consequently, these results suggest that comprehensive nutritional support should not only provide general dietary advice but also incorporate specific guidance to enhance supplement literacy. Even for supplements commonly used by athletes, appropriate guidance is necessary to prevent unnecessary consumption if scientific evidence on efficacy is limited. Furthermore, considering that athletes are easily influenced by their immediate social circles (24), supplement education should be extended not only to the athletes themselves but also to coaches and support staff. This approach would ensure a consistent, evidence-based information environment for the athletes as well as those around them.

This study has some limitations. First, data collection relied on self-reported information, which introduces potential recall bias and reporting inaccuracies. Although registered dietitians verified supplement details through interviews, the knowledge and practices regarding hydration and supplementary foods remained self-assessed rather than objectively validated. In addition, the lack of data on the frequency, dosage, and duration of supplement use precludes a precise quantitative assessment, providing only a broad overview of prevalence. Addressing these factors in future research is essential to evaluate the practical significance of athletes' supplementation patterns. Second, the questionnaire used in this study was originally developed for clinical use in routine medical checkups and was not formally validated for research purposes. Therefore, the reliability and validity of the instrument, including aspects such as construct validity and test-retest reliability, were not established. This may have introduced measurement bias and may limit the comparability of our findings with those obtained using validated instruments, such as the Nutrition for Sport Knowledge Questionnaire (33) and other previously developed tools for assessing supplement use (26). Furthermore, the data are primarily descriptive and do not fully capture detailed behaviors related to supplement intake. Taking these factors into consideration, future research may need to use standardized tools. Third, the definition of nutritional support in this study includes one-off seminars and regular individual counseling; however, the frequency, content, and degree of individualization of support are unclear. Thus, it cannot be ruled out that differences in supplement use may exist between athletes who have only attended seminars and who receive regular individual counseling. Fourth, certain limitations regarding our study population should be noted. There was a substantial imbalance in the sample size between the groups with and without nutritional support experience (86.9% vs. 13.1%), which may have limited the statistical power of the comparisons and affected the stability of some estimates in the logistic regression. Additionally, because the findings are based exclusively on Japanese elite athletes, cultural, dietary, and organizational differences in athletes' support systems between countries may limit the generalizability of these results to international athletic populations. Finally, one participant consumed a supplement that could not be classified according to the AIS. This supplement was excluded from the calculation of supplement intake rates. Given that only one supplement could not be classified and that few athletes consumed this supplement, substantial changes to the study results are unlikely.

In this study, a total of 92.3% of Japanese athletes and candidates for the 33rd Olympic Games (Paris/2024) reported using one or more supplements during the previous year. Athletes with nutritional support experience were more likely to use supplements, particularly sports foods. Even after adjusting for age and sex, an association was found between sports food consumption and nutritional support experience. However, these results were insufficient to confirm a distinct difference in group C supplement intake between the two groups, suggesting the requirement for further investigation. These findings suggest that the nutritional support experience is associated with the prevalence of overall supplement intake, whereas it may not be clearly linked to the use of supplements with limited scientific evidence. Future studies should focus on disseminating scientific evidence and safety information about supplements to athletes. Management by registered dietitians involved in athlete support plays a critical role in nutritional care. Collaboration with physicians and pharmacists is essential for educating athletes on the appropriate use of supplements.

## Data Availability

The data analyzed in this study is subject to the following licenses/restrictions: The datasets analyzed during the current study are not publicly available due to ethical and privacy restrictions concerning the elite athlete participants, but are available from the corresponding author on reasonable request. Requests to access these datasets should be directed to Akiho Shinagawa, shinagawa6114@gmail.com.
